# Implementation of TomoEDGE in the independent dose calculator CheckTomo

**DOI:** 10.1002/acm2.12048

**Published:** 2017-03-06

**Authors:** Mathieu Schopfer, Simon J. Thomas, G. Samuel J. Tudor, Jean Bourhis, François Bochud, Raphaël Moeckli

**Affiliations:** ^1^ Institute of Radiation Physics CHUV and University of Lausanne CH‐1007 Lausanne Switzerland; ^2^ Department of Medical Physics Addenbrooke's Hospital Cambridge CB2 0QQ UK; ^3^ Department of Radio‐Oncology CHUV and University of Lausanne CH‐1007 Lausanne Switzerland

**Keywords:** independent, quality assurance, tomotheraphy

## Abstract

**Purpose:**

CheckTomo is an independent dose calculation software for tomotherapy. Recently, Accuray (Accuray Inc., Sunnyvale, CA, USA) released an upgrade of its tomotherapy treatment device, called TomoEDGE Dynamic Jaws, which improves the quality of treatment plans by enhancing the dose delivery with the help of jaws motion. This study describes the upgrade of CheckTomo to that new feature.

**Methods:**

To account for the varying width and off‐axis shift of dynamic jaws fields, the calculation engine of CheckTomo multiplies the treatment field profile by a penumbral filter and shifts the dose calculation grid. Penumbral filters were obtained by dividing the edge field profiles by that of the corresponding nominal field. They were sampled at widths 1.0, 1.8, and 2.5 cm at isocenter in the edges of the 2.5 and 5 cm treatment field.

**Results:**

The upgrade of CheckTomo was tested on 30 patient treatments planned with dynamic jaws. The gamma pass rate averaged over 10 abdomen plans was 95.9%, with tolerances of 3 mm/3%. For 10 head and neck plans, the mean pass rate was 95.9% for tolerances of 4 mm/4%. Finally, misplacement and overdosage errors were simulated. In each tested cases, the 2 mm/3% gamma pass rate fell below 95% when a 4 mm shift or 3% dose difference was applied.

**Conclusions:**

These results are equivalent to what CheckTomo achieves in static jaws cases. So, in terms of dose calculation accuracy and errors detection, the upgraded version of CheckTomo is as reliable for dynamic jaws plans as the former release was for static cases.

## 1 Introduction

Independent dose verification is considered to be important to ensure patient safety.^1^ It can be performed through an independent calculation with commercial softwares for three‐dimensional conformal radiation therapy (3DCRT), image‐modulated radiation therapy (IMRT), and volume‐modulated arc therapy (VMAT) treatments. For tomotherapy, as far as we know, there exists a commercial tool, Mobius 3D (Mobius Medical Systems, Houston, TX, USA), and a single‐point dose verification software.^2^ Additionally, an open source solution, CheckTomo, was released in 2011.^3^ That software independently generates a three‐dimensional point‐based dose distribution, using patient CT images and delivery plan, and compares it against the dose volume calculated by the tomotherapy treatment planning system (TPS).

Accuray (Accuray Inc., Sunnyvale, CA, USA) released an upgrade of its tomotherapy device called TomoEDGE Dynamic Jaws.^4^ The purpose of this upgrade is to reduce the field penumbra along the patient longitudinal (inferior–superior) axis by the mean of jaws motion. The way the dose is delivered is hence modified and the dose calculation engine of CheckTomo needed to be upgraded consequently.

This study aims to present the work done to develop and implement the upgrade of CheckTomo and the tests that were performed to assess that the dose calculation carried out with the upgrade is as reliable as it was with the previous version. It does not suggest any improvement of the core calculation engine.

## 2 Materials and methods

### 2.A TomoEDGE dynamic jaws

In tomotherapy, the field is delimited in the longitudinal (IEC‐y) direction by a pair of collimators, called jaws. A non‐TomoEDGE direct or helical tomotherapy treatment is delivered with static jaws, i.e., at fixed field width during the whole treatment procedure, either 1, 2.5, or 5 cm at isocenter. This implies that the field penumbra in the longitudinal direction is of approximately the field size on both cranial and caudal sides of the target. To limit the extra dose to organs at risk (OAR) and other healthy tissues, the treatment can be delivered with a smaller field width, but this usually increases the irradiation time.

**Figure 1:**
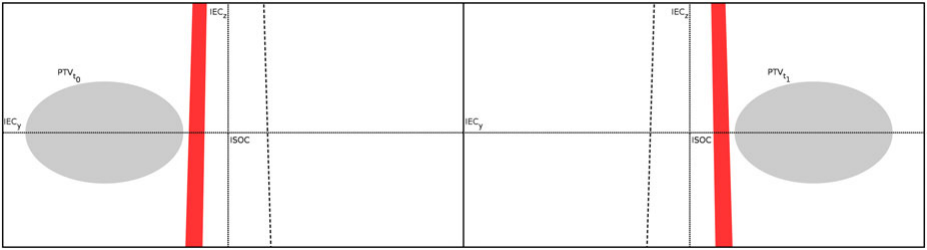
Schematic representation of a TomoEDGE treatment beam at two moments. Dashed lines represent the nominal field width. Edge fields (in red) are represented at treatment start (right) and end (left). At treatment start, the jaws delimit a 1 cm wide field on the negative IEC‐y side of the beam axis. During treatment (not represented), as the PTV moves forward, the cranial jaw opens to keep the superior field edge ahead of the PTV superior limit. Then, the caudal jaw closes to keep the inferior field edge behind the PTV inferior limit. Finally, when the treatment ends, the jaws delimit a 1 cm wide field again, but on the positive IEC‐y side of the beam axis.

To overcome this poor trade‐off, TomoEDGE introduced jaws motion during treatment delivery.^5^ At treatment start, the jaws delimit at isocenter an asymmetrical 1 cm wide field, located off the source axis toward the patient's feet. Then as the couch moves forward, the cranial jaw sweeps toward the patient's head to keep the field edge 5 mm ahead of the planning target volume (PTV), until the jaws delimit a symmetrical field (respectively to the beam axis) of the nominal treatment size, either 2.5 or 5 cm at isocenter. Similarly, the caudal jaw closes behind the PTV as it exits the beam, until the field is 1 cm wide again.^4^ In a TomoEDGE treatment, the penumbra on the cranial and caudal sides of the PTV is reduced to 1 cm. See Fig. 1 for a graphical depiction.

For clarity, the fields will be denominated “nominal” when delimited by symmetrically positioned static jaws and “edge” otherwise.

### 2.B. CheckTomo

#### 2.B.1 Software basics

CheckTomo is a software written in MATLAB (The MathWorks Inc., Natick, MA, USA) that computes a three‐dimensional point‐based dose distribution using CT data and treatment plan on the patient side and independently acquired beam data on the machine side.

Patient data are read from DICOM CT and RT‐plan files where beam geometry and patient position during treatment are described.

Beam data are provided with CheckTomo for each nominal treatment field in text files with a homemade structure. They consist of a reference dose point, tissue‐phantom ratios (TPRs), output factors (OFs), and off‐axis ratios (OARs) measured for various field shapes. The 5 × 40 cm^2^ field at isocenter was taken as the reference one and the dose reference point was measured isocentrically at depth 10 cm. All machine data were independently acquired on a tomotherapy unit using an ionization chamber at different depths in a water tank.

CheckTomo dose distribution is usually calculated on a grid of 15 × 15 × 15 points, with a 1 to 1.5 cm spacing. Grid resolution and size can be adapted if needed. For each sinogram projection (or control point), the dose deposited at a particular location is the product of the projection time, the dose rate, TPR, OF, and OAR. The fluence is considered to arise from the mean angle of the projection arc, which, regarding the tomotherapy standard of defining 51 control points per gantry rotation, extends over 7.29°. To increase the number of control points and thus improve the dose calculation accuracy, CheckTomo offers the option to split each projection into multiple subprojections.^6^

CheckTomo dose distribution can be compared to that calculated by the tomotherapy treatment planning system (TPS) by means of a gamma^7^ or box comparison index.^8^ Required patient data, beam data collection, dose calculation model, and comparison indices were explained in more detail in the original release paper of CheckTomo.^3^

#### 2.B.2 Beam profile model

In CheckTomo, the longitudinal profile of a nominal field is calculated by multiplying the field OAR, the TPR, and the OF. CheckTomo handles OARs expressed in angular distance respectively to the beam source, instead of Cartesian coordinates. It follows the tomotherapy naming conventions of field size, calling the longitudinal dimension the width and the in‐plane dimension the length (width and length are always given at isocenter). Which is more, the OF of the tomotherapy beam, hereafter *S*_cp_, is not a function of the equivalent square field size but depends independently on both the field width and length.^3^ In CheckTomo, it is therefore considered to be a function $S_{\mathrm{cp},w_{0}}$ of the field length specific to the nominal field of width *w*_0_.

Thus, the longitudinal profile at angular coordinate $\theta_{y}$ and depth *d* of a nominal field of width *w*_*0*_ and length *L* is given by

(1) $$P_{N}\left(w_{0},L,\theta_{y},d\right)={\text{OAR}}_{y}\left(\theta_{y},d\right)\cdot \text{TPR}\left(A_{\mathrm{sq}},d\right)\cdot S_{\mathrm{cp},w_{o}}\left(L\right).$$

*A*_sq_ is the equivalent square field size.

### 2.C. Implementation of a dynamic jaws beam profile model in CheckTomo

Jaws motion induces changes in the field shape and OF that have to be accounted for in the profile model. Theoretically, the longitudinal profile of an edge field is obtained by multiplying Eq. (1) by a jaw penumbral filter and by correcting the OF. But as mentioned in section 2.B.2, the OF function *S*_cp_ was not designed to account for a varying field width. To overcome this limitation, the relative jaw penumbral filter (RJPF) was introduced, defined as the ratio of the edge and nominal longitudinal profiles P_E_ and P_N_,

(2) $$\text{RJPF}\left(w,w_{0},\theta_{y},d\right)=\frac{P_{E}\left(w,w_{0},L,\theta_{y},d\right)}{P_{N}\left(w_{0},L,\theta_{y},d\right)}.$$

Here P_E_ is the edge field profile given in angular coordinates respectively to the beam source. The transformation consists in first applying a coordinates shift along the longitudinal axis so that the field maximum is at IEC−y = 0. Then, the shifted Cartesian coordinates are converted in angular distances.

The edge field profile equation is obtained by inverting relation (2) and replacing P_N_ with equation (1), namely

(3) $$\begin{array}{cc} P_{E}\left(w,w_{0},L,\theta_{y},d\right)= & \;{\text{OAR}}_{y}\left(\theta_{y},d\right)\cdot \text{TPR}\left(A_{\mathrm{sq},d}\right)\cdot S_{\mathrm{cp},w_{0}}\left(L\right)\\ & \cdot \text{RJPF}(w,w_{0},\theta_{y},d). \end{array}$$

Note that it yields a profile originating at the source axis. To account for the edge field off‐axis nature, the dose calculation grid is shifted longitudinally — toward head or feet depending on the edge side — by half the field width.

In practice, P_E_ and P_N_ were sampled at field widths and depths specified in section 2.D, normalized, respectively, to P_N_ peak maxima and converted into angular coordinates. RJPFs were then calculated from Eq. (2) by interpolating P_E_ and P_N_ over for a set of arbitrary points. These data were stored in new text files structured like the existing CheckTomo beam data files. Note that the RJPFs were not sampled at different field lengths because it was checked that this parameter only has a slight influence on the longitudinal profiles. Lastly, for widths and depths falling between sampling values, the RJPF is interpolated on the fly at run time.

### 2.D. Measurements of edge beam data

Profiles measurements were performed with an Exradin A1SL ionization chamber (Standard Imaging, Middleton, WI, USA) in a water tank at SSD 85 cm, all MLC leaves open and depths 1.5, 5, 10, 15, and 20 cm. They were all run successively for the nominal and edge fields.

Measurements of both the edge and nominal profiles were needed to calculate the RJPF from Eq. (2). The edge field width varying continuously between 1 cm and the nominal field size, it was necessary to pick some sampling values. During the TomoEDGE acceptance test procedure (ATP), field data were measured for widths 1.0, 1.8, and 2.5 cm in both edges of 5 cm nominal field. We decided to perform profile measurements for that same set of values. Due to the flattening filter free (FFF) beam of tomotherapy units, the profile of an edge field depends also on its distance to the source axis. So, similar measurements were performed in the edge of the 2.5 cm nominal field as well. Obviously, it was sufficient to realize them only on one side of the source axis.

### 2.E. Dose calculation verification and tests of accuracy

#### 2.E.1 Gradient check

Five plans were generated using the images of the Cheese Phantom and the 5.0 cm plans structures set provided with the TomoPhant IMRT verification patient, which is usually available in the tomotherapy TPS. Three PTVs of 2 cm, 6 cm, and 10 cm were created by shrinking or extending the original target volume. Plans were calculated for the 2.5 cm field on these three PTVs and for the 5 cm field on the 6 cm and 10 cm PTVs. All plans were calculated in dynamic jaw mode. The PTVs were centered on the machine isocenter, the prescription dose was of 2 Gy and the pitch was 0.287. To force some field modulation, a constraint was applied on a structure of the same size as the target located 2 cm beneath it.

All five plans were calculated in CheckTomo with a 2.5 mm longitudinal spacing and global 2 mm/3% and 3 mm/4% gamma indices were calculated. Additionally, the dose profiles along the longitudinal axis in the isocenter plane were extracted from both the CheckTomo and tomotherapy TPS dose volume so that they could be compared visually.

**Table 1:** Gamma pass rate (γ) for two tolerances and average mean dose difference ($\bar{\Delta D}$) of the five plans calculated in the TomoPhant. Points within the 50% isodose and at least 5 mm depth were considered in the calculation of the gamma index

Field width [cm]	PTV length [cm]	γ 2 mm, 3% [%]	$\overset{¯}{\gamma }$ 3 mm, 4% [%]	$\bar{\Delta D}$ [%]
2.5	2	91.2	96.4	2.2
2.5	6	100.0	100.0	0.0
2.5	10	99.8	100.0	−1.0
5.0	6	88.0	96.0	−0.1
5.0	10	86.7	95.3	−0.9

**Table 2:** Geometrical setup, gamma pass rate (γ) for various tolerances and average mean dose difference ($\bar{\Delta D}$) of the 10 abdomen plans. Points within the 50% isodose and at least 5 mm depth were considered in the calculation of the gamma index

Case	PTV dose [Gy]	Field width [cm]	PTV length [cm]	γ 2 mm, 3% [%]	γ 3 mm, 3% [%]	γ 4 mm, 4% [%]	$\bar{\Delta D}$ [%]
A01	2	5.054	30.2	97.8	99.2	100.0	−0.9
A02	1.8	2.51	29.2	83.7	88.6	96.4	−1.8
A03	2	2.51	9.75	100.0	100.0	100.0	0.27
A04	1.8	2.51	17.6	97.0	98.2	99.2	−0.5
A05	7	2.51	3.8	97.5	99.1	100.0	1.93
A06	1.8	5.054	20.4	84.2	89.6	94.5	1.38
A07	2	2.51	13	99.5	99.8	100.0	−0.2
A08	2	2.51	12.2	83.6	85.8	93.0	2.56
A09	3	2.51	19.2	97.9	98.6	99.7	−0.8
A10	2.3	2.51	33.4	99.6	99.9	100.0	−0.7

#### 2.E.2 Dose verification in real patient cases

The upgrade of CheckTomo was tested on 30 patient cases planned and treated with dynamic jaws. All plans had successfully passed a clinical quality assurance (QA) test which consisted in comparing the TPS dose distribution to a measurement performed with an Octavius 729 detector array in an Octavius II phantom (PTW, Freiburg, Germany). Dose comparison was done in VeriSoft (PTW, Freiburg, Germany) using a 3 mm/3% gamma comparison index^7^ for points within the 10% isodose and considering a 95% pass rate threshold.

The independent calculation of the dose distributions was performed with the upgraded version of CheckTomo using the original patients CT images, a 31 × 31 × 31 calculation grid with a longitudinal spacing of 6 mm (8 mm in two cases) and one subprojection per projection. These grid settings ensured us to cover in each case a major part of the PTV and to get a reasonably high dose point resolution in the field edges. Note that in some cases, the PTV was too large to fit entirely in the dose calculation grid. PTV length and field width for each patient are given in Tables 2, 3, 4.

**Table 3:** Geometrical setup, gamma pass rate (γ) for various tolerances and average mean dose difference ($\bar{\Delta D}$) of the 10 head and neck plans. Points within the 50% isodose and at least 5 mm depth were considered in the calculation of the gamma index

Case	PTV dose [Gy]	Field width [cm]	PTV length [cm]	γ 2 mm, 3% [%]	γ 3 mm, 3% [%]	γ 4 mm, 4% [%]	$\bar{\Delta D}$ [%]
HN01	2	2.51	12.75	69.2	74.6	88.9	3.7
HN02	2.12	2.51	7.75	65.6	76.6	89.6	3.1
HN03	2.12	2.51	14.75	100.0	100.0	100.0	0.3
HN04	2.12	2.51	15	88.1	91.8	97.9	2.3
HN05	2	2.51	12.4	90.0	92.5	97.5	2.2
HN06	2.12	2.51	15.6	93.5	96.1	98.5	1.6
HN07	2	2.51	13.8	92.3	93.3	96.8	0.9
HN08	2	2.51	11.2	75.0	82.0	90.6	3.0
HN09	2.12	2.51	16	95.2	96.7	99.0	0.8
HN10	2.12	2.51	18.75	99.2	99.3	100.0	0.1

**Table 4:** Geometrical setup, gamma pass rate (γ) for various tolerances and average mean dose difference ($\bar{\Delta D}$) of the 10 breast plans. Points within the 50% isodose and at least 5 mm depth were considered in the calculation of the gamma index

Case	PTV dose [Gy]	Field width [cm]	PTV length [cm]	γ 2 mm, 3% [%]	γ 3 mm, 3% [%]	γ 4 mm, 4% [%]	$\bar{\Delta D}$ [%]
B01	2	2.51	23.6	91.5	95.2	98.2	2.0
B02	2	2.51	14.4	73.4	81.8	91.5	2.8
B03	2	2.51	21.8	87.4	92.6	96.8	2.0
B04	1.8	2.51	21.4	98.1	99.1	99.7	1.3
B05	2	2.51	25.4	95.8	97.0	99.7	1.5
B06	2	2.51	24.6	98.2	98.8	99.7	0.5
B07	2	2.51	20.6	90.2	93.7	97.8	2.0
B08	2	2.51	20.4	85.1	91.0	96.5	2.4
B09	2	2.51	23.8	96.3	97.3	99.3	1.1
B10	2.65	2.51	20.2	71.1	88.2	91.7	2.8

**Table 5:** Number of successes to the gamma comparison test ($N_{\gamma >95\%}$, i.e., pass rate above 95%) and mean gamma pass rate ($\overset{¯}{\gamma }$) for various tolerances for the three different regions investigated. Ten treatment plans were tested in each region. Points within the 50% isodose and at least 5 mm depth were considered in the calculation of the gamma index

	Abdomen and pelvis		Head and neck		Breast	
	$N_{\gamma >95\%}$	$\overset{¯}{\gamma }$ [%]	$N_{\gamma >95\%}$	$\overset{¯}{\gamma }$ [%]	$N_{\gamma >95\%}$	$\overset{¯}{\gamma }$ [%]
2 mm, 3%	7	94.1	3	86.8	4	88.7
2 mm, 4%	7	97.2	7	93.1	5	92.3
3 mm, 3%	7	95.9	4	90.3	5	93.5
3 mm, 4%	8	97.9	7	95.0	8	94.9
3 mm, 5%	10	98.9	7	97.5	9	98.0
3 mm, 6%	10	99.4	10	98.5	10	98.9
4 mm, 4%	8	98.3	7	95.9	8	97.1
$\bar{\Delta D}$ [%]	1.1		1.8		1.8	

The calculation accuracy was assessed for each of the 30 plans by computing the mean dose difference and performing global gamma comparison tests between the CheckTomo and the tomotherapy TPS dose distributions. The gamma index was calculated for various tolerances over the points located within the 50% isodose and at least 5 mm deep in the patient's body. A test is considered successful if its gamma pass rate is above 95%.

#### 2.E.3 Errors simulation

Finally, in order to test the ability of the upgrade of CheckTomo to detect errors, 15 cases that had passed a 2 mm/3% gamma comparison test were selected, independently of the treatment location. Then longitudinal misplacements and overdosages were simulated over them by applying a 2 mm and 4 mm coordinate shift and a 3% dose offset to the TPS dose distribution.

## 3 Results and discussion

### 3.1 Edge beam data and profiles model

**Figure 2:**
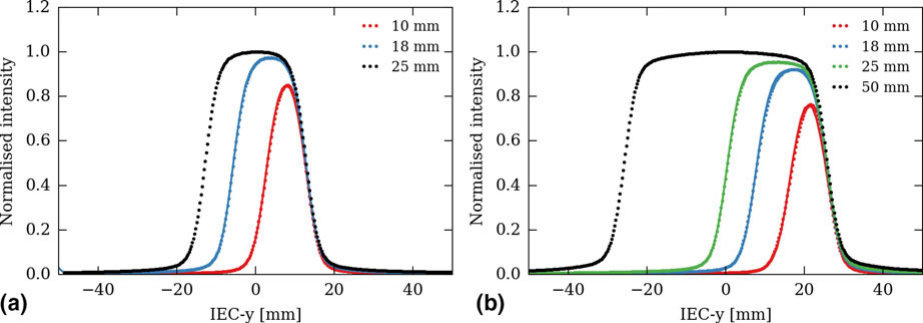
Longitudinal profiles, along the machine IEC‐y axis, of the 2.5 cm (a) and 5 cm (b) on‐axis nominal fields (black) and their related off‐axis edge fields (colored). Dots represent measurements, plain lines the edge profiles calculated from Eq. (3). Measurements were performed at 1.5 cm depth, all leaves open, on the positive IEC‐y side of the gantry. Values in the legend correspond to the field width at isocenter.

Figure 2 shows field profiles measured and calculated on the positive IEC‐y side of the gantry. All profiles were normalized to the maximum of the corresponding nominal field. The difference in relative intensity between a profile in the edge of the 2.5 cm and 5 cm nominal field is visible, particularly for the 1 cm field.

Note that Eq. (3) yields a symmetric approximation of the edge field profiles, which are actually asymmetric (because the position of the jaws compared to the axis of the beam generates asymmetric penumbra). This approximation is inherent to the beam model of CheckTomo, which was not designed to handle asymmetric fields. Though, as can be seen in Fig. 2, the calculated edge profiles (plain lines) show a good agreement with the measurements (dots). The maximal error induced by the approximation of Eq. (3) is of respectively 2.7% and 1.5% for the 1 cm and 2.5 cm edge fields. Also note that in Eq. (3), the spatial coordinate is the angular distance at the source, but that the field profiles are represented in Fig. 2 in Cartesian coordinates.

**Figure 3:**
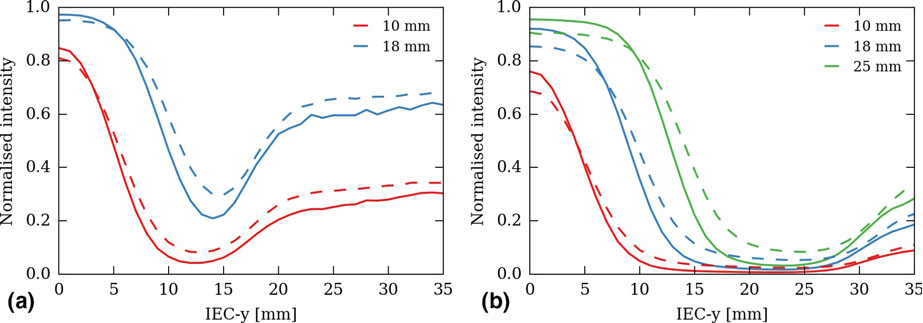
Relative jaw penumbral filters of the 2.5 cm (a) and 5 cm (b) nominal fields for each of the three off‐axis edge field widths sampled, along the machine longitudinal (IEC‐y) axis. RJPF are shown at depth 1.5 cm (solid lines) and 10 cm (dashed lines).

Figure 3 shows the relative jaw penumbral filters of the 2.5 cm and 5 cm nominal field, defined by Eq. (2) and calculated using the measured profiles shown in Fig. 2. One can see that the RJPFs are depth‐dependent, as are the field profiles. Also, one should note that they do not converge toward 0 when reaching the field limit, as would be expected. This is a numerical artifact: obviously, both the nominal and edge field profiles also tend toward 0 at the field boundary, and dividing two small values one with another [in Eq. (2)] may result in large numbers. In other words, the RFJPs are hardly calculable outside the field. Though, this is not an issue because the product of the profile and the RJPF [in Eq. (3)] converges toward 0 at the field limit. As can be seen on Fig. 2, the calculated edge field profiles (plain lines) match the measurements (dotted lines).

Finally, CheckTomo upgrade was designed having in mind that TomoEDGE could in the future evolve and perform more complex dose sculpting. One can think of sharpening the edges of a simultaneous integrated boost (SIB) or tracking a tumor.

### 3.B Dose calculation gamma pass rate

#### 3.B.1 Gradient verification

Gamma index pass rates for all five plans calculated in the TomoPhant are given in Table 1. With the 2.5 cm field, the pass rate is high (99.8%) for the 6 cm and 10 cm target. For the 2 cm target, the index tolerance must be increased to 3 mm/4%. Note that this case was designed for testing purposes. In clinical practice, it would not make sense to try to cover a 2 cm long PTV with the 2.5 cm wide field and the 1 cm field would have been used instead.

**Figure 4:**
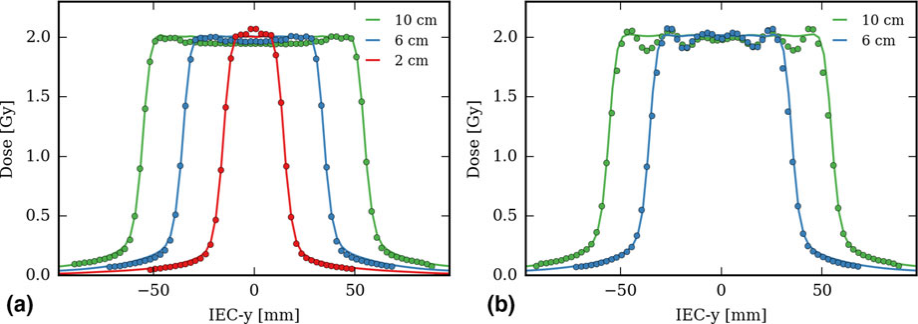
Dose profiles of the plans calculated in the TomoPhant for varying PTV length, for the 2.5 cm field width (a) and 5 cm (b). Plain line corresponds to the dose calculated by the tomotherapy TPS and dots to CheckTomo dose points.

The gamma pass rates of the plans calculated with the 5 cm field are lower, below 90% for the 2 mm/3% tolerance. As can be seen in Fig. 4 (b), the dose calculation is perturbed over 5 cm by the approximation of the varying field width profile. Though, this figure also shows that calculation of the field gradient by CheckTomo matches well that of the TPS both in space and dose.

#### 3.B.2 Real patient cases and errors detection

The calculation of 29,791 dose points for one case takes between 2 and 3 minutes on Intel Core i5 3.4 GHz processor, depending on the size of the region of interest considered.

CheckTomo was tested on 10 abdomen and pelvis, 10 head and neck (H&N), and 10 breast plans. For each case, the mean dose difference ($\bar{\Delta D}$) and global gamma comparison tests between the CheckTomo and the tomotherapy TPS dose distributions were calculated. Individual results of all cases are provided in Tables 2, 3, 4. For each location and tolerances, the number of plans that succeed the gamma test ($N_{\gamma >95\%}$, i.e., pass rate above 95%) and the mean gamma pass rate ($\overset{¯}{\gamma }$) over the 10 plans are given in Table 5. One can see that plans in the abdominal and pelvic region are the most accurately calculated with at least 7 plans out of 10 succeeding the gamma comparison test. In the opposite, dose calculation for the H&N cases is more prone to errors and requires the gamma index dose tolerance to be increased to 4% to have a majority of plans succeeding the test. This can be explained by the fact that PTVs in the abdominal and the pelvic area usually encompass large homogenous tissue volumes, while bones and air cavities can be found in the H&N region. The difference of calculation accuracy between those two kinds of location comes from the fact that the dose calculation in CheckTomo relies on a water‐based model, which is obviously more reliable in tissues with densities close to water. Note that the scope of this manuscript is to describe the implementation of TomoEDGE in CheckTomo, not to suggest improvements of its calculation engine.

Concerning the breast cases, where the target volumes are often off‐axis, increasing the number of subprojections per projection from 1 to 3 or 5 could improve the results accuracy.^6^

Table 6 shows the results of the error simulation tests. It concerns 15 cases that had passed the 2 mm/3% gamma comparison test. As one can see, all plans failed the 2 mm/3% gamma test when a 4 mm shift was applied longitudinally to the calculation grid or if the dose was offset by 3%.

**Table 6:** Number of cases succeeding the gamma test ($N_{\gamma >95\%}$), mean pass rate ($\overset{¯}{\gamma }$), and average mean dose difference ($\bar{\Delta D}$) for 15 treatment plans on which was applied a longitudinal shift of 2 and 4 mm and a dose offset of 3%. Only plans which had passed (without simulated error) a 2 mm/3% gamma test were considered

	Unshifted	2 mm shift	4 mm shift	Overdosage 3 %
$N_{\gamma >95\%}$ [%]	15	13	0	0
$\overset{¯}{\gamma }$ [%]	98	96.7	83.7	63.2
$\bar{\Delta D}$ [%]	0.79	0.77	0.91	3.49

The results of the gamma comparison tests presented here for dynamic cases are similar to what had been obtained for static jaws plans with the original release of CheckTomo.^3^ In other words, the overall dose calculation accuracy and sensitivity to errors is equivalent for both TomoEDGE and non‐TomoEDGE plans.

Performing an independent dose calculation with CheckTomo is not as comprehensive as actually measuring it during a QA procedure, in that sense that it performs no control on the machine side. Though, CheckTomo successfully detected simulated errors exceeding tolerances. In other words, it is conservative of the quality assurance, thus can provide a good indicator of the accuracy of the dose calculation. Nonetheless, the way CheckTomo could be used in practice (e.g., replace a patient QA measurements) remains the responsibility of the local medical physicist.

### 3.C Occasional edge dose calculation error

**Figure 5:**
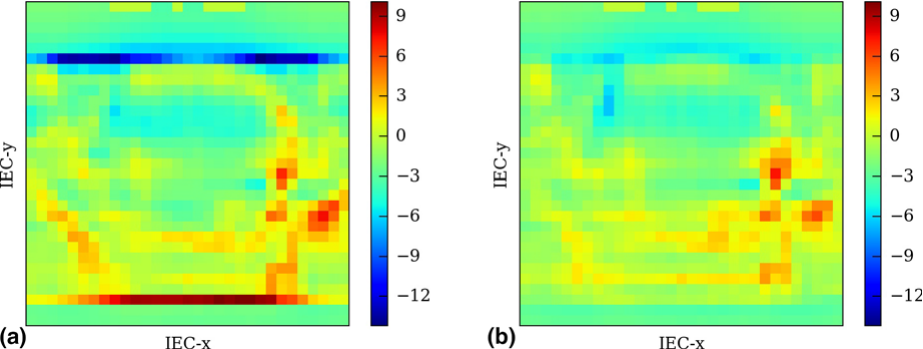
Coronal view of the relative difference, given in percent, between the CheckTomo and the tomotherapy TPS dose of a pelvis plan. As one can see in figure (a), the dose is miscalculated in both edges of the target volume. The error is corrected by applying a 1 mm shift in the longitudinal direction (IEC‐y) to the TPS dose distribution, as shown in figure (b).

In some cases, the dose is over or under estimated in the target volume edges, as shown in Fig. 5 left‐hand side. The occurrence of such errors seems random and is caused by rounding mistakes in the calculation of the dose grid coordinates. Even a submillimetric registration error between the CheckTomo and tomotherapy TPS dose distributions could lead to a dose miscalculation of several Gy within the high gradient region. Though, such a problem can be easily addressed by shifting longitudinally the TPS dose volume, using a manual registration tool included in CheckTomo since the first version. As it happens, the error appearing in Fig. 5 was corrected by applying a 1 mm shift. The result is shown on the figure right‐hand side.

Even if such an error is not accounted for, it does not much impact the overall gamma pass rate of the plan (0.3% in the case of Fig. 5). The relative dose difference does usually not exceed 10% and concerns only the points located in the field edges, hence a small portion of the PTV. However, one should note that CheckTomo was not specifically designed to be a dose gradient verification tool and should not try to use it as such. CheckTomo cannot isolate a particular region of interest and lacks analysis tools dedicated to conformality verification.^9^

## 4 Conclusion

CheckTomo software for independent dose calculation in tomotherapy was upgraded for TomoEDGE treatments by introducing the RPJF in its profile calculation model. It was noted that this method implies that a slight inaccuracy in the edge field profiles calculation has to be tolerated. The results of the gamma comparison tests demonstrated that, in terms of dose calculation accuracy and errors detection, the upgraded version of CheckTomo is as reliable for dynamic jaws plans as the former release was for static cases. This leads us to conclude that, from now on, CheckTomo offers the opportunity to perform independent dose calculation equivalently for both static and dynamic jaws tomotherapy plans.

## Conflict of interest

R. Moeckli is holding a grant from Accuray for a research project in tomotherapy. However, the present work is not directly related to that grant.
